# Seismic loading of fault-controlled fluid seepage systems by great subduction earthquakes

**DOI:** 10.1038/s41598-019-47686-4

**Published:** 2019-08-05

**Authors:** Marco Bonini

**Affiliations:** 0000 0001 1940 4177 grid.5326.2 CNR, Consiglio Nazionale delle Ricerche, Istituto di Geoscienze e Georisorse, Sede Secondaria Firenze, via G., La Pira 4, 50121 Firenze, Italy

**Keywords:** Natural hazards, Geodynamics

## Abstract

Various types of fluid expulsion features (mud volcanoes, pockmarks, authigenic carbonate mounds and associated gas pipes, etc.) are often found above subduction zones, which have the highest seismic potential on Earth. Faults potentially control the liberation of deep-seated greenhouse gases into the feeder systems of seepage features located above subduction thrusts. These feeder systems could be stressed by large earthquakes, yet the mechanisms that can drive episodic mobilization of stored hydrocarbon gases remain poorly understood. Here I address the potential stress loading on fluid expulsion systems created by past earthquakes nucleated at both accretionary and erosive subduction margins. The most significant effects occur in the epicentral area where subduction earthquakes can produce normal stress changes as high as 20–100 bar, although these are generally restricted to relatively small regions. Coseismic normal stress changes and elastic strain relaxation upon a ruptured subduction thrust could increase crustal permeability by dilating fault-controlled conduits, and channelling fluids to the seafloor. Fluid pressure pulses released during subduction earthquakes can greatly contribute to the rupture of fluid pathways that have been brought closer to failure from coseismic static stress changes, although the inaccessible location of most submarine seepage systems has so far hampered probing these relationships.

## Introduction

Methane and other greenhouse gases are released from submerged margins of subduction zones through a variety of fluid seepage systems including mud volcanoes, authigenic carbonate mounds, calderas, pockmarks and associated gas pipes/chimneys that may be ultimately controlled by directivity through faults^1–5^. Mud volcanoes are steep-side conical edifices produced by the extrusion of subsurface mud breccia, water, and gases (dominantly methane)^1,6^. Such constructional edifices can grow up to a few hundred meters in height and extend for several square kilometers. Submarine mud volcanoes can grow to even larger dimensions, and develop in accretionary wedges driven by upwelling gaseous hydrocarbons (e.g. Mediterranean Ridge, Makran, Barbados, etc.)^1^, or abiogenic methane produced by the serpentinization of peridotite, as for the gigantic mud volcanoes of Marianas^7^.

Other common seepage structures are cold methane seeps hosting various biological communities, which may favour the precipitation of authigenic carbonates creating positive-relief carbonate mounds^2^. Fluid and gas leakage can also produce negative-relief features, such as pockmarks and caldera depressions. Mud volcanoes and other methane seepage structures are often sourced through complex feeder systems that may exploit faults and reach depths of some kilometres^5,7–9^. Activity of submarine fluid expulsion features is likely to be episodic, similar to terrestrial mud volcanoes that release large amounts of greenhouse gases during eruptive events that may occasionally interrupt their quiescent activity^10,11^. These submarine methane seepage features are often (but not invariably) linked to the subduction of oceanic crust, and collectively represent an important input of methane and other hydrocarbons into the hydrosphere^1,6,7^.

Subduction zones have the ability to generate the most powerful earthquakes on Earth (up to >M9), and such events have been shown to trigger the massive release of hydrocarbons. For example, a significant increase in upward flux of gas-hydrate-derived methane ensued in a few decades after the (M_w_8.1) 1945 Makran earthquake^12^. Also submarine mud volcanoes show a sensitivity to earthquakes^13^, and therefore conceivably behave as those subaerial, for which a connection with eruptions and earthquakes has been documented^10,11,14,15^. Nevertheless, eruptions or increased activity of deep-sea mud volcanoes and fluid expulsion structures are only seldom reported^16,17^ and consequently triggering of these features by subduction megathrust earthquakes has been basically disregarded to date. Therefore, the processes governing the variation in gas flux emitted from submarine seepage features, as well as the relationships with the megathrust earthquake cycling, are poorly known.

**Figure 1:**
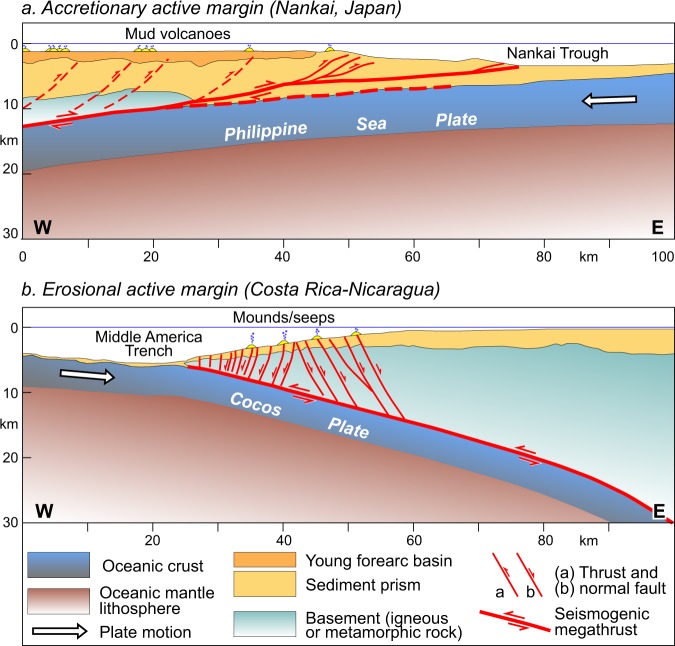
Conceptual cross sections showing structure of fault-controlled methane seepage at convergent plate boundaries. (**a**) In accretionary active margins, methane is frequently released at mud volcanoes, which are sourced from thrust faults splaying off from the subducted plate interface (Nankai subduction zone; adapted from Park *et al*.^30^). (**b**) In erosional active margins, methane is often released via normal faults that die out near the basal subduction interface and form seabed seepage structures such as pockmarks and authigenic carbonate mounds and associated gas pipes (Costa Rica-Nicaragua subduction zone; adapted from Ranero *et al*.^18^). This figure was generated using Adobe Illustrator CS3 (https://www.adobe.com).

One route to an understanding of fluid seepage features’ behaviour is the estimation of the potential loading of past subduction earthquakes on these systems. Transient dynamic stresses produce large shaking of fluid expulsion systems, which would lead to an increase in reservoir pressure^14,15^. Coseismic static stress changes are generally much smaller than dynamic stresses, but are permanent and may be exceptionally large at mud volcanoes located above subduction zones. The reason for this behaviour is twofold: (1) the extremely large magnitude of megathrust earthquakes, and (2) the structural position of seepage structures and their deep plumbing systems, which are often controlled by faults rooted into the subduction thrust^8,9,18^. Static stress would thus play an important role also during post-seismic periods, particularly the normal stress changes could act in a way to open or close the fault-controlled fluid pathways (see Methods). To investigate the role of such stress changes, I have explored the potential loading produced by recent subduction earthquakes on fault-controlled conduits, even though the variations over time of fluid discharge is rarely known^16,17^. The consideration that fault-controlled conduits generally extend at depth near the subduction thrust (Fig. 1) has motivated the choice of a modelling that involves an elastic and isotropic rheology (see Methods).

## Submarine Fluid Seepage Systems at Subduction Zones

Seismic activity and slippage along the subduction interface is accomplished by a subduction channel, which also collects overpressured waters and gases (mostly thermogenic methane and heavier hydrocarbons)^19^. For temperatures >150 °C the subduction channel gets progressively locked and becomes the nucleation area of ruptures generating great intraplate earthquakes^18,19^. Two main types of subduction margins can be identified, particularly (i) accretionary and (ii) erosive^20^. Accretionary and erosive margins bear distinctive structural settings that may control the type of fluid seepage structures and the propensity to generate mega-earthquakes or tsunami earthquakes^21^. In accretionary margins the incoming sediments are scraped off and accreted into the accretionary prism of the upper plate, whereas in erosive margins the sediments are subducted or underplated beneath the overriding plate. Consequently, at accretionary margins the prism is deformed internally by active thrusts producing ridges sub-parallel to the trench axis. Erosive margins show a more complex behaviour as they are characterised by reverse faulting in the frontal part of the wedge^22^, while large normal faults may develop more inland consequently to the subsidence of the upper plate caused by removal of material from its base^18^.

Two end-members of methane release are considered, namely those controlled by thrusts and normal faults in accretionary and erosive subduction margins, respectively. Normal faults driving fluids to the sea bottom may also result from predominantly horizontal extension ensuing in the overriding plate during large subduction earthquakes^5,23^. Notably, both types of fault-controlled fluid pathways have been documented to reach the subduction fault zone and convey upward fluids causing seafloor hydrocarbon seepage^5,8,9,18,24^. Coseismic normal stress changes acting permanently on faults and fluid conduits have been inferred to promote or discourage coseismic and post-seismic fluid flow (and eruptions) in magmatic^25–27^ and mud volcano^11^ systems. On this basis, this study focuses on normal stress changes that have been resolved on thrust or normal faults, channelling putative deep sourced liquids, gases and solid material upwards. The setting of methane-dominated seepage systems representative of accretionary and erosive subduction margins is examined in the sections below.

## Accretionary Margins. Nankai Subduction Zone, Japan

**Figure 2:**
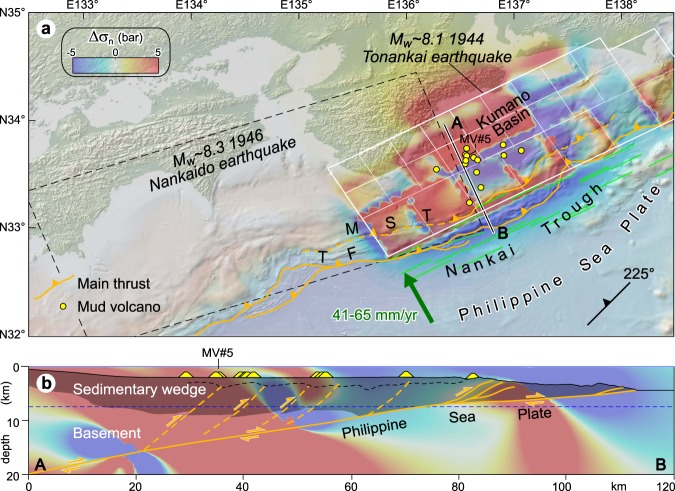
(**a**) Normal stress changes (Δσ_n_; bar, fault unclamping positive) produced by the 1944 Tonankai earthquake (finite fault model from Baba *et al*.^32^; see also Fig. S1 and Supporting Information). Seismic source (white box), rectangular sub-faults (white cells) and their surface projection (green lines) are indicated. Small yellow circles indicate mud volcanoes in the Kumano Basin (after Pape *et al*.^6^; Morita *et al*.^29^). The Kumano Basin is almost entirely located above the 1944 fault rupture. Stress changes are resolved on thrust faults (strike = 225°, dip = 40°, rake = 90), which are inferred to control the mud volcano systems in the Kumano Basin. The rupture area of Kato & Ando^31^ for the 1946 Nankaido earthquake (dashed black line) is shown for reference. TF, thrust front; MST, megasplay thrust. (**b**) Vertical cross section of normal stress changes superposed onto the Nankai subduction margin (simplified from Park *et al*.^30^). Both the sedimentary wedge (indicated by gray shading) and the underlying ‘basement’, which collectively indicates dominant igneous and metamorphic rocks (island arc upper crust; Marcaillou *et al*.^70^), are indicated. Deep-sourced fluids emitted from mud volcano MV#5 are inferred to travel upward along a thrust fault rooted in the subduction interface^8^. A convenient range of stress change values (−5, 5 bar) has been arbitrarily chosen; note that in some models the calculated stresses may exceed this range. In horizontal section a stress is sampled at 7.5 km depth (dashed blue line in b). This figure was generated using the Coulomb 3.4 software (https://earthquake.usgs.gov/research/software/coulomb) and Adobe Illustrator CS3 (https://www.adobe.com).

Northwest-directed subduction of the Philippine Sea Plate beneath the Eurasian plate occurs offshore southern Japan at a rate of ~4.1–6.5 cm yr^−1^, forming the Nankai Trough^28^. Submarine mud volcanoes occur on the Kumano forearc basin topping the inner Nankai accretionary prism^29^. The Nankai accretionary prism is deformed by a major landward dipping thrust fault splaying from the subduction plate boundary and surfacing at the deformation front of the wedge^30^. This megasplay thrust is proposed to have ruptured in 1944 beneath the Kumano Basin, generating the M_w_ ~ 8.1 Tonankai earthquake, and to the southwest two years later generating the M_w_ ~ 8.3 1946 Nankaido earthquake^31^ (Fig. 2a). At least 14 mud volcanoes 100–200 m in height and 1–2 km in diameter have been identified in the Kumano Basin. These mud volcanoes are sourced from shallow anticlines in the accretionary prism through a subvertical plumbing system connecting the anticlines crests to the seafloor^29^. Some of these anticlines are associated with ancient thrust faults splaying off from a main décollement layer^24^. Nishio *et al*.^8^ have shown that fluids currently expelled at mud volcano MV#5 originate from a deep (∼15 km) seismogenic fault connecting the mud volcano to the basal décollement (Fig. 2b). There are therefore strong indications that thrust faults in the accretionary wedge are the main structural pathways channelling deep overpressured fluids to mud volcano systems^8,24^. Based on average fault trend and dip^24^, normal stresses changes produced by the Tonankai earthquake have been calculated on thrust faults with strike 225°, dip 40° and rake 90°. Stresses are sampled in different positions along a 40°-dipping thrust, particularly (1) at the surface, (2) 5 km (i.e., an approximate thermogenic gas production depth^6^) and (3) 12–15 km, which is the source depth of some fluids probably along a pressurized fluid-bearing basal décollement^8^.

Normal stress changes acting on these faults are evaluated using six finite fault models, which results are reported in Fig. S1 (see Supporting Information). The great majority of finite fault models employ source faults deeper than the subduction thrust inferred from seismic reflection data (Fig. S1a–e). The model by Baba *et al*.^32^ is instead the one that best fits this seismic profile-based subduction thrust (Fig. S1f), even though in the horizontal section this solution appears less smooth than the others, as observed from distribution of stress at the edges of sub-faults. This may depend on cell dimensions, as large cells can produce stress concentrations; however, in cross section the stress pattern is rather smooth (Fig. S1f). In general, the six finite fault models show some variations, but basically give similar stress change patterns, with the majority of mud volcanoes being located in positive (red) normal stress change patches (Fig. S1). Nevertheless, stress change magnitude depends critically on the vicinity to the source fault. Considering mud volcano MV#5 (see above), the highest values are obtained from Baba *et al*.’s^32^ model (Fig. 2a), which yields stress changes varying from ~3 bar (seafloor) to ~38 bar (15 km depth), in the vicinity of the seismogenic subduction thrust (Fig. 2b). The other source faults yield smaller normal stress changes (~0.7–12 bar; Table S1), since they are located at distance from the interpreted subduction thrust (Fig. S1).

## Erosive Margins. Costa Rica-Nicaragua

Subduction of the Cocos plate beneath the Costa Rica-Nicaragua continental margin is accomplished by tectonic erosion of the overriding plate, which is undergoing extension and subsidence^18,33^. Methane-derived seepage structures mainly consist of pockmarks and authigenic carbonate mounds^2^, which are often structurally controlled by seaward dipping normal faults stretching the erosive wedge^18^. Nearly 130 seepage structures have been identified along the subduction margin^18,22^, yet this number is likely underestimated.

**Figure 3:**
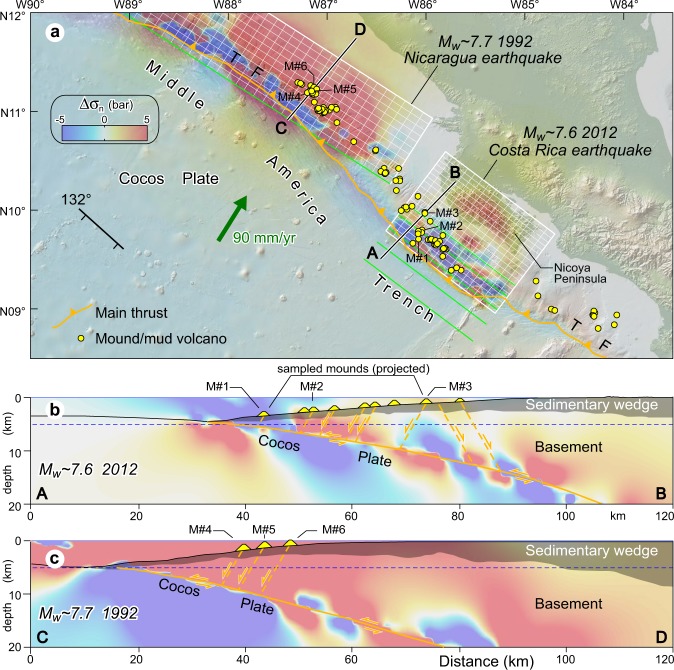
(**a**) Normal stress changes (Δσ_n_; bar, fault unclamping positive) created by the 2012 Costa Rica and 1992 Nicaragua earthquakes (finite fault models from Hayes^34,36^; symbols are as those in Fig. 2); see also Fig. S2 and Supporting Information. Small yellow circles indicate different types of seafloor methane seeps (after Ranero *et al*.^18^ and Kluesner *et al*.^22^). (**b**,**c**) Vertical cross sections of normal stress changes superposed onto the Costa Rica^18^ and Nicaragua^33^ wedges (sedimentary wedge in gray shading). The emitted fluids are inferred to travel upward along normal faults rooted in the subduction interface, and thus stress changes are resolved on seaward-dipping normal faults (strike = 132°, dip = 60°, rake = −90°). A convenient range of stress change values (−5, 5 bar) has been arbitrarily chosen; note that in some models the calculated stresses may exceed this range. In horizontal section a stress is sampled at 5 km depth (dashed blue line in vertical cross sections b and c). This figure was generated using the Coulomb 3.4 software (https://earthquake.usgs.gov/research/software/coulomb) and Adobe Illustrator CS3 (https://www.adobe.com).

On 2012 September 5, the subduction thrust ruptured beneath the Nicoya Peninsula, producing a M_w_7.6 earthquake (Fig. 3a). This epicentral region is located southeast of the thrust segment that ruptured offshore Nicaragua on September 2, 1992, generating a M_w_7.7 earthquake. Both rupture areas are located beneath many methane seepage structures (Fig. 3a). Normal stress changes have been resolved on receiver normal faults (strike 132°, dip 60° and rake −90°) using the available source fault models for the 2012 and 1992 earthquakes (Fig. 3a and S2).

With regard to the M_w_7.6 2012 earthquake, the source faults of Hayes^34^ and Yue *et al*.^35^ fit best the subduction thrust reported in Ranero *et al*.^18^, although there is a slight mismatch between these faults at 15–20 km depth (Fig. S2). The patterns of normal stress changes produced by this earthquake are rather complex, with positive and negative patches variously distributed according to the considered finite-fault rupture model (see Fig. S2 and Table S2). Depending on their position, the feeder systems of some seepage structures have been clamped, while others are unclamped and brought closer to failure. In order to understand the magnitude of normal stress changes, I have considered the loading on some normal faults controlling methane seepage structures along cross section AB (Fig. 3a,b; Table S2). Stresses are sampled at the surface and near the intersection of a 60° dipping fault with the basal thrust décollement. In the considered cases, normal stress changes are mostly positive and yield a maximum value of ~33 bar at mound M#1 near the subduction thrust. Normal stress change estimated for M#2 are mostly positive (~2–10 bar), while for M#3 normal stress changes are positive (~3 bar) and negative (~2 bar) depending on the source model (Table S2).

As regards the M_w_7.7 1992 Nicaragua earthquake, the source fault by Hayes^36^ is practically coincident with the subduction thrust reported in Ranero *et al*.^33^ (Fig. 3c). Interestingly, the great majority of seeps fall within a broad unclamping region (Fig. 3a). Normal stress changes have been extracted from the numerical models and sampled at discrete positions along the inferred fault-controlled conduit of some mounds (M#4–6) located along section CD (Fig. 3a,c). Normal stress changes estimated for these mounds are invariably positive (unclamping) and range between ~8–15 bar at the sea bottom and ~7–42 bar near the intersection with the subduction thrust (Table S2).

## Discussion

### Effect of normal stress changes on crustal permeability

I have evaluated above the effect of coseismic static stress changes produced by mega-earthquakes on thrust and normal faults serving as channels in the present-day methane seeps located above subduction thrusts. Normal stress change patterns are complex and distribution of positive and negative patches vary greatly depending on the considered finite fault model (Figs 2, 3, S1 and S2). Coulomb stress changes show similar complex patterns. In particular, reverse and/or normal faults coexist in the region above the ruptured subduction thrust, and these faults are brought closer or farther from failure according to their position^37^.

Both thrust (Nankai margin) and normal faults (Costa Rica-Nicaragua margin) are favourably oriented for being opened by normal stress changes when falling in an unclamping stress area. In magmatic systems, relatively small earthquake-induced unclamping stress changes have been considered influential in favouring volcanic eruptions by dilating structurally–controlled magma ascent paths, such as for the eruptions of Vesuvius (~0.1‒0.2 bar)^25^, Karymsky (~0.5 bar)^26^, and Copahue (~1.5‒2.5 bar)^27^. Unclamping normal stress changes resolved on the fault-controlled fluid pathways considered in this study yield even higher values, particularly ~1‒38 bar for Nankai thrusts and ~2‒42 bar for normal faults offshore Costa Rica-Nicaragua (Figs 2 and 3, Tables S1 and S2). Stress changes computed so far have been produced by earthquakes with M_w_ ~8, and obviously the magnitude of these changes can potentially increase largely for more powerful plate-boundary earthquakes.

**Figure 4:**
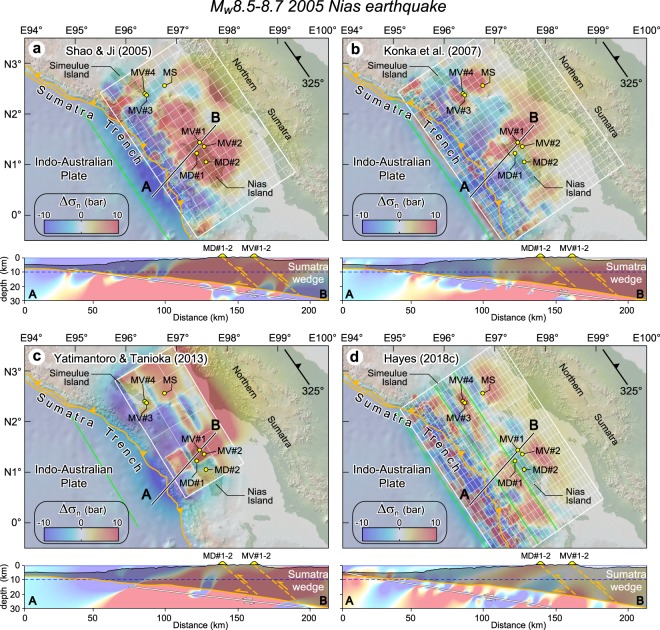
Normal stress changes (Δσ_n_; bar, fault unclamping positive) produced by the M_w_ ~ 8.5–8.7 2005 Nias earthquake considering different finite fault models: (**a**) Shao & Ji^39^, (**b**) Konca *et al*.^40^, (**c**) Yatimantoro & Tanioka^41^, and (**d**) Hayes^42^ (symbols are as those in Fig. 2). A depth cross section is shown in the bottom panel of each source fault model. Small yellow circles indicate different types of subaerial and seafloor methane seeps. Normal stress change patterns are superposed onto the Sumatra accretionary wedge (the latter is indicated by gray shading), which includes undifferentiated igneous and sedimentary rocks, as well as a mantle wedge and slivers of oceanic crust (adapted after Singh *et al*.^43^). A convenient range of stress change values (−10, 10 bar) has been arbitrarily chosen; note that in some models the calculated stresses may exceed this range. The thick white lines in cross sections indicate the source fault considered in the respective finite fault model. In horizontal sections stress is sampled at 10 km depth (dashed blue line in vertical cross sections). This figure was generated using the Coulomb 3.4 software (https://earthquake.usgs.gov/research/software/coulomb) and Adobe Illustrator CS3 (https://www.adobe.com).

For instance, the Sumatra subduction zone has recently experienced giant earthquakes and is a region characterized by mud volcanism^38^. In particular, the M_w_8.5–8.7 Nias earthquake of March 28, 2005 ruptured the subduction thrust beneath northern Sumatra forearc, an area where mud volcanoes and mud diapirs are exposed on Nias and Simeulue islands^38^, and a cold methane seep is documented in the offshore forearc basin (Fig. 4 and Table S3). Normal stress changes have been resolved on thrust faults (strike 325°, dip 40° and rake 90°) that are exposed on the islands using the finite fault models of Shao & Ji^39^, Konca *et al*.^40^, Yatimantoro & Tanioka^41^, and Hayes^42^. Stress changes are calculated up to a depth of 20 km, which corresponds approximately to the intersection of thrusts with the subducted plate interface assumed as that inferred to have generated the M_w_ > 9 December 26, 2004 Sumatra earthquake^43^. Interestingly, the inferred thrust-controlled fluid pathways are predominantly unclamped, with normal stress changes locally exceeding 50 bar at depth in areas above highest slip gradients (Fig. 4; Table S3). However, the various source faults show some misfit with the Sumatra subduction thrust^43^ (see section AB in Fig. 4), possibly because it refers to a section located more than 250 km to the north. Normal stress changes calculated near the source faults yields negative and positive values that may locally even exceed 100 bar (Table S3). It is however unknown whether such stress changes have promoted eruptions or increases in fluid flow.

The upper plate is pervasively affected by thrust or normal faults, which generally bear tensile strength lower than intact rocks. Tensile rock strength estimated in laboratory tests is around and usually less than 10 MPa (=100 bar)^44^, but this magnitude may vary over a broader range (ca. 0.2–20 MPa) depending on the rock type and the integrity of the rock mass^45^. More importantly, laboratory tests addressing the tensile strength of geological discontinuities (joints, veins, and bedding), which may be representative of fault-controlled fluid conduits, generally yield levels on the order of 0.5–2 MPa (5–20 bar)^46^. The above results collectively suggest that suitably high normal stress changes generated by great subduction megathrust earthquakes can locally attain values similar or even in excess of tensile strength of rock discontinuities, especially nearby the ruptured subduction thrust (Tables S1–S3). These stresses may reopen, or at least may contribute to dilate existing fault-controlled fluid pathways and increase crustal permeability above ruptured subduction thrusts.

However, the computed normal stress changes do not support ubiquitous tensile failure over the whole area because they can overcome the tensile strength of existing discontinuities only locally (i.e., within regions with sufficiently high positive normal stress change). Where normal stress changes are negative, these would instead shrink the conduits producing conditions unfavorable for permeability enhancement. In this regard, it is worth noting that relaxation of the viscoelastic mantle after a subduction earthquake induces significant tension in the overlying elastic upper plate^47^, which is also consistent with the fore-arc extensional aftershocks that followed the M_w_8.8 Maule and partly the M_w_9.0 Tohoku earthquakes^23,37^. Whereas normal stress change-related increased permeability would be limited to positive normal stress patches, relaxation in the elastic strain over the ruptured thrust has the potential for increasing permeability over larger regions of the upper plate. It is however likely that both mechanisms interact and contribute to increase crustal permeability.

### Factors promoting fluid pressurization and flow during large subduction earthquakes

The passage of seismic waves causes dynamic stresses inducing massive ground shaking and a variety of hydrologic responses including fluid flow and coseismic permeability increase^48^. In the near field region, the associated peak dynamic stresses can easily exceed 40 bar and are of the same order of magnitude of static stresses^49,50^, even though the latter decay with distance *R* from the epicenter as 1/*R*^3^, thereby much faster than dynamic stress changes decreasing as 1/*R*^1.66^ (e.g., ref.^51^). In mud volcano and other pressurized systems, the transient dynamic stress changes can generate overpressures in the fluid source or in the mud itself according to various mechanisms, such as liquefaction, incoming low frequency seismic waves, amplification and focusing of seismic energy at parabolic seismic reflectors, etc.^14,15^. Seismic waves have also the ability to increase permeability by flushing and clearing particles from clogged discontinuities^52^ or opening fractures^53^. Earthquakes can also breach the seals separating pressurized compartments (fluid reservoirs), producing an enhanced permeability, fluid flow, and redistribution of pore pressures, with high pore pressures diffusing across the breached barrier^51,54–56^. Consequently, fluids captured in pressurized compartments along subduction thrusts are released through the breaking of hydraulic barriers during large intraplate earthquakes, generating postseismic fluid flow^57^.

**Figure 5:**
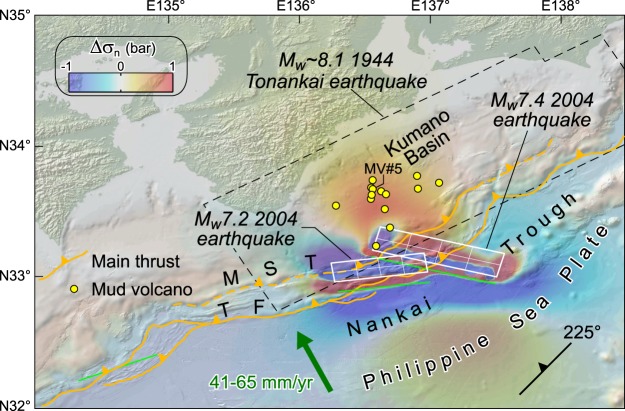
Normal stress changes (Δσ_n_; bar, fault unclamping positive) created by the M_w_7.2 and M_w_7.4 earthquakes of 05 September 2004. Source faults of 2004 earthquakes are based on focal mechanism solutions available from USGS (https://earthquake.usgs.gov/earthquakes/eventpage/usp000d3ka/executive; https://earthquake.usgs.gov/earthquakes/eventpage/usp000d3mb/executive), and fault dimensions hinge on the empirical relationships of Wells & Coppersmith^69^; stress is sampled at 7.5 km depth. The  rupture area of Baba *et al*.^32^ for the 1944 Tonankai earthquake (dashed black line) is shown for reference only. Symbols are as those in Fig. 2. This figure was generated using the Coulomb 3.4 software (https://earthquake.usgs.gov/research/software/coulomb) and Adobe Illustrator CS3 (https://www.adobe.com).

The above stress computations have shown that, in some regions of the upper plate, coseismic normal stress changes have the ability to produce extensional stresses sufficiently high to exceed the tensile strength of rock discontinuities. Dilatation of structurally-controlled pathways, together with elastic strain relaxation, can thus favour eruption or increase the degassing of seepage systems, and deliver massive quantity of pressurized, deep-sourced fluids into the seawater. For instance, mud volcano MV#5 in the Kumano basin enhanced its methane emission over a 6-year period subsequent to the M_w_7.2 and M_w_7.4 earthquakes of 05 September 2004^17^, which unclamped the inferred thrust-controlled fluid conduit by only ~0.5 bar (Fig. 5; Table S1).

Deep-seated reservoirs of pressurized fluids (mud and dissolved hydrocarbon gases) and the overlying feeder system can be viewed as incompressible systems that may be perturbed by both static and transient dynamic strain. Volumetric strain estimated for the 1944 Tonankai earthquake is dilatative (up to +10^−5^) for five source models out six (Table S1). A similar result is obtained for the 2012 Costa Rica and 1992 Nicaragua earthquakes, for which a dilatational strain field is estimated with the exception of one source model (Table S2). Both volumetric expansion^58^ and compression^25^ of magma reservoirs have been advocated for initiating eruptions or increasing fluid release. Assuming a linear poroelastic material with factors *B* = 1 and *K*_u_ = 6 GPa, the strains reported in Tables S1 and S2 imply pore pressure changes less than ~0.15 bar (see Methods), with the majority of them producing pore pressure decreases. Assessing the effect of the sign of volumetric strain is difficult for fluid seepage settings, yet, if expansion is really a viable mechanism to increase fluid discharge, then the estimated volumetric strains would play some role in this process.

### Seismic cycling and implication for the liberation of greenhouse gases

Regional tectonic stress and smaller scale variations in the stress field can be other important players, as documented for instance on the western Svalbard margin where active fluid release coincides with regions of tensile stress regime^59^. In subduction zones, tectonic stress is dictated by the megathrust cycling. The recognition that the 2010 Maule earthquake produced coseismic extensional faulting along the Chilean margin, led Geersen *et al*.^5^ to hypothesise that the seismic cycle of a given subduction thrust segment would modulate periodic gas discharge. This concept could also be extended to other fluid expulsion systems including mud volcanoes, even though terrestrial mud volcanoes show a limited sensitivity to earthquakes, the majority of eruptions being not driven by earthquakes^10,14^. On the other hand, marine seepage systems are intimately linked to subduction zones, which represent the most continuous and powerful seismogenic structures on Earth. It is thus possible that seismic triggering is more important for marine rather than for terrestrial mud volcanoes and other fluid seepage systems, although detection of submarine eruptions has hitherto been hampered by the deepwater location of these features.

Under the hypothesis that the seismic cycle modulates seepage system hydrology, fluids would be released periodically during coseismic and early-post-seismic stages as a result of coseismic normal stress changes and relaxation in the elastic strain upon a ruptured fault. Horizontal tectonic compression, characterizing the inter-seismic period^23^ is instead interpreted to hinder fluid flow by closing the fault-controlled conduits, an evolution also assisted by self-healing of fault processes. Fluid release may even cease when the characteristic recurrence time of subduction earthquakes is sufficiently long and fault healing processes efficient. Erosive subduction margins (like Costa-Rica-Nicaragua) would experience mainly extension during the inter-seismic stage, thereby opposing fault closure. Fluid discharge at various methane seepage features is thus a complex process depending on different factors that may change with time. More specifically, periodic fluid seepage would be modulated by both internal factors (rate of natural fluid/gas recharge, fault healing state, etc.) and external forcing, particularly the seismic cycling of the underlying subduction thrust, or the superimposition of more recent static stresses created by rupture of contiguous thrust segments.

### Potential failure mechanisms assisting upward migration of fluids

It is well known that in fluid-saturated seismogenic crust, stresses, fluid pressure, and fault strength at failure remain intrinsically linked together, and can control the modality of stress loading of faults^60^. Fluid pulses produced during great subduction earthquakes^57^ represent an important component of the system, which may impact heavily on the mode of fault loading. Coseismic release of pulses of high fluid pressure originated from a main shock plane can indeed trigger aftershocks on adjacent faults. Also fossil systems record the ability of fluid pressure pulses generated from rupture of regional structures to trigger neighbouring small-displacement faults, as evidenced by distribution of fault-related ore deposits that strikingly match distributions of aftershocks predicted through Coulomb stress change modelling^61^. However, the magnitude of pressure pulses may largely exceed that of static stress changes, and can trigger aftershocks even in regions where Coulomb stress changes would discourage faulting^55^.

**Figure 6:**
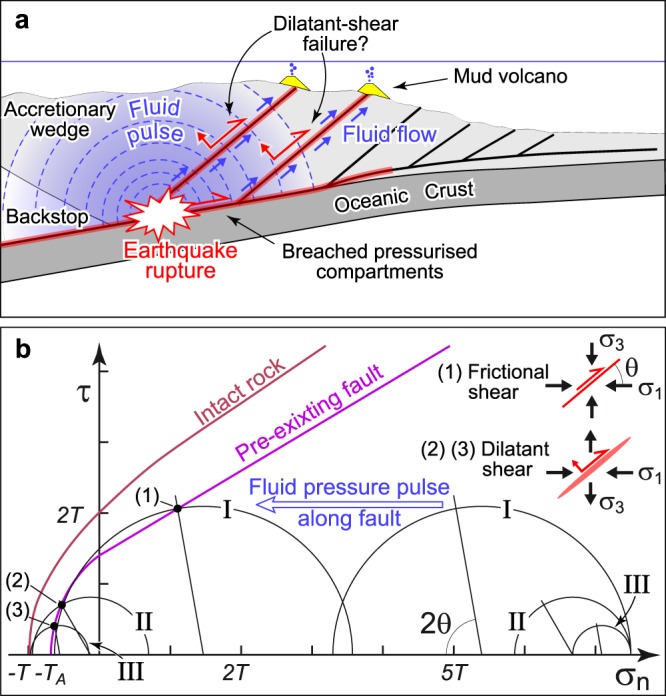
Conceptual model showing the inferred relationships between pressure pulses originated from subduction earthquakes and failure of pre-existing thrust faults in an accretionary margin. (**a**) Breaching of pressurised sectors along the ruptured subduction thrust induces pulses of overpressured crustal fluids channelled along adjacent, and steeper splay thrusts. (**b**) Composite Griffith-Coulomb failure envelope representing the shifting of initial stress states by a fluid pressure pulse localising fluid flow up-through a fault. The modality of failure depends on the differential stress magnitude. Half circles I and III represent different stress states required for producing frictional shear (I) and dilatant shear failure (III) on a 40°-dipping thrust fault. Dilatant shear failure on a 40°-dipping thrust needs a very small differential stress (half circle III), while a slightly larger stress state is necessary for a 30°-dipping thrust fault (half circle II). Both half circles II and III require a minimum principal stress lower than tensile strength of anisotropic material (*T*_*A*_), a condition that can be maintained only transiently (i.e., until when fluid pressure pulse overcomes fluid pressure loss caused by tensile fracturing). This figure was generated using Adobe Illustrator CS3 (https://www.adobe.com).

Given these observations, it is proposed that fluid pressure pulses released by earthquakes on the subducted plate interface could migrate up through adjacent and steeper thrust faults owing to their higher permeability with respect to wall rock (Fig. 6a). Besides causing aftershocks, such a fluid pulse-driven fault weakening contributes to the rupture of faults that may have been already brought closer (or even farther) to failure from coseismic static stress changes. Under these conditions, fault-controlled pathways may fail according to frictional shear failure or dilatant shear failure depending upon differential stress magnitude. This behaviour is schematically illustrated in Fig. 6b through a Mohr-Coulomb diagram showing failure envelope for both intact and anisotropic (fault weakness) material. The considered 40°-dipping thrust is rather misoriented for dilatant shear failure, which requires very low differential stress and the support of a stress state beyond the failure envelope for tensional fracturing of anisotropic material (half circle III in Fig. 6b). A similar condition is also necessary for a 30°-dipping thrust (or a 60°-dipping normal fault), although differential stress is slightly larger (half circle II). These conditions can be achieved transitorily in case of extreme fluid pressure pulses^62^, during which dilatant shear faulting may initiate together with tensile fracture of pre-existing vein margins along the fault. Fault-controlled fluid pathways would thus become transiently permeable and focus fluid flow during repeated failure events that are conceivably modulated by seismic cycling. Dilatant shear failure would represent an efficient mechanism for transferring hydrocarbon fluids stored either along the main subduction plane or in deep overpressured reservoirs (Fig. 6a).

Interseismic wedge compression yields to thrust fault loading and differential stress increase, favouring frictional shear failure (e.g., half circle I in Fig. 6b). Accretionary wedges are usually characterised by undrained and fluid-saturated conditions, and frictional shear failure can occur at substantially lower shear strengths owing to poroelastic load weakening behaviour taking place for thrusts dipping ≤45° ^62^. For a 40°-dipping thrust settled in fluid-saturated rocks, the increase in fluid pressure needs be ~0.4 of stress increase^62^.

## Conclusions

Subduction megathrust earthquakes produce multiple effects on mud volcano and other methane seepage systems, namely (1) coseismic shaking and pressurization/depressurization of fluid reservoirs by dynamic and static strains, (2) coseismic and early post-seismic opening/closing of fault-controlled fluid channels, and (3) fluid pulses released from ruptured overpressured compartments. In particular, subduction earthquakes can induce relaxation in the elastic strain above the ruptured thrust and transfer massive static stress changes to thrust and normal faults, which provide fluid channel networks in the upper plate. Any subduction zone on Earth is potentially associated with stored methane, a combination that poses a strong, global natural hazard regarding the liberation of fossil carbon that may be triggered by the expected increase in crustal permeability created by mega-earthquakes. Although only gases released from shallow submarine seeps (generally <500 m water depth) have the potential to reach the atmosphere^63^, the methane dissolved in seawater is frequently oxidised by microbial activity into carbon dioxide that in turn can reach the atmosphere and/or increase ocean acidification^64^.

Megathrust earthquake cycling could influence the release of deep-stored fossil fluids, with massive input of greenhouse gases in the hydrosphere/atmosphere during coseismic and early-post-seismic periods. Seismically triggered release of methane can thus represent an important component of the global carbon budget. However, the sensitivity of marine fluid expulsion systems to earthquakes depends on a number of intrinsic factors, and also the connections between subduction earthquakes and eruptions are often overlooked because these features are dominantly located underwater. On the other hand, episodic fluid seepage may also occur during slow slip events along basal subduction thrusts, as proposed for the Hikurangi subduction zone^65^.

The mechanisms by which the release of deeply-stored fluids occur is difficult to determine because of the complexity and inaccessibility of the subsurface. More work and information are therefore needed to test the correlation between the predicted stress change patterns and areas with increased post-seismic fluid discharge. A future challenge will consist in the long-term monitoring of fluid emissions over subduction zones (i.e., pre- and post-rupture fluid discharge) to probe the factual response of seepage systems to static stresses changes. Despite the simplifying assumptions of the numerical modeling, this study spotlights the potential importance of coseismic normal stress changes for assessing the mechanisms controlling fluid expulsion along subduction margins.

## Methods: Static Stress Changes and Volumetric Strain

Fault interaction is often investigated by calculating the Coulomb stress changes induced by an earthquake fault rupture on the surrounding crust. Stress on ‘receiver’ faults nearby the earthquake rupture is permanently changed as a function of their position, geometry and sense of slip (rake). Specifically, a certain receiver fault will fail if the applied stress increment, defined by the Coulomb Failure Function^66–68^, rises above a stress threshold specific for the considered fault. The Coulomb Failure Function, ΔCFF^66–68^ is defined as:

1 $${\rm{\Delta }}\mathrm{CFF}={\rm{\Delta }}{\rm{\tau }}+{\rm{\mu }}\,{({\rm{\Delta }}{\rm{\sigma }}}_{{\rm{n}}}+{\rm{\Delta }}p),$$

where Δσ_n_ is the fault-normal stress change (positive if the fault is unclamped), Δτ is the shear stress change (positive in the direction of fault slip), Δ*p* is the pore pressure change within the fault, and μ is the static friction coefficient. Equation (1) is often indicated in its simplest form, ${\rm{\Delta }}\mathrm{CFF}={\rm{\Delta }}{\rm{\tau }}+{\rm{\mu }}^{\prime} {\rm{\Delta }}{{\rm{\sigma }}}_{{\rm{n}}}$, where μ′ is the apparent coefficient of friction that contains the coefficient of friction (μ), the pore-pressure change and the material properties of the fault zone. Increased shear stress changes (Δτ) and unclamping of faults (Δσ_n_) can encourage failure of receiver faults, with fault friction modulating the role of fault unclamping.

The ΔCFF is normally used to determine whether the static stress changes promote or discourage slip on faults, and therefore earthquake-induced static stress changes acting on potential fault-controlled fluid pathways have been calculated together with volumetric static strain in crust immediately after a large rupture. In particular, this study focuses on the role of normal stress changes in opening fault-controlled fluid pathways, though also the other static stresses are considered. Specifically, Coulomb Failure Function, shear stress changes and normal stress changes caused by a subduction earthquake have been resolved at different depths on specific receiver fault-controlled fluid conduits, which are defined by their strike, dip, and rake, though rake does not influence the normal stress change (all stress changes are reported in Tables S1–S3 of Supporting Information).

Most studies find that static stress changes play a significant role in the triggering of aftershocks and following mainshocks on contiguous faults^37,66–68^. Stresses are computed using a three-dimensional elastic half-space boundary element model (Coulomb 3.4 software; https://earthquake.usgs.gov/research/software/coulomb) with uniform isotropic elastic properties, as detailed in King *et al*.^68^. The calculations of static stress changes on fluid pathways have been carried out using published finite-fault rupture models (part of which are available at the online SRCMOD database accessible at http://equake-rc.info/srcmod), or the source fault dimensions have been determined using the Wells & Coppersmith’s^69^ empirical relations. In particular, large earthquakes have complex slip distributions that can be constrained inverting various data (i.e., teleseismic body waves, tsunamis waveforms, InSAR data, GPS vectors). These data are used to define a ‘finite fault model’, in which the source fault is discretized into small rectangular cells that represent independently rupturing ‘sub-faults’, each of which has a specific slip vector and can be considered to be an earthquake. Number and dimensions of cells depend on the finite fault model. Finite fault models are thus based on different methods testing the correspondence between specific physical features measured at different seismic stations or localities, and those predicted from the considered source fault model (e.g., synthetic seismic waveforms). Although reliability assessment of published source models is beyond the purpose of this study, a first approximation estimate of model reliability can be inferred from how well the modelled source fault fits the actual fault geometry imaged from available seismic reflection profiles or other geophysical data (see also main text).

The pattern of static stress changes depends on the finite fault rupture model, as well as the geometry, rake and friction coefficient of the surrounding receiver faults. Spatial distribution of static stress changes also depends on the magnitude of different input parameters, such as the Poisson’s ratio, the Young’s modulus and the apparent coefficient of friction. Unfortunately, an accurate magnitude of these parameters for the specific study cases is poorly known, thereby in these computations I have assumed average values. In particular, the upper crust has been modelled using uniform values for elastic moduli, namely: Poisson’s ratio *ν* = 0.25 and Young’s modulus *E* = 80 GPa, based on King *et al*.^68^. A value of μ′ = 0.4 has been assumed as an average of the 0.0–0.8 range of possible values of the apparent coefficient of friction^67,68^; higher or lower values of μ′ would slightly enhance or reduce the effect of static stress changes^68^. Similarly, an increase or a decrease of the Young’s modulus would enhance or diminish the magnitude of stress changes, respectively. For simplicity, the same Young’s modulus and Poisson’s ratio have been assumed for all the examined study cases. Although natural systems are likely characterized by heterogeneities and weakness zones, detailed information about subsurface structure and material properties are lacking, and thus I assume homogenous elastic properties. These parameters (i.e., Poisson’s ratio, the Young’s modulus and the apparent coefficient of friction) may increase or decrease the effect of static stress changes, and may thus affect the magnitude and spatial distribution of small scale stress change variations. As stated earlier, the magnitude of these parameters is generally poorly known, and it is thus important to emphasize that the obtained stress change patterns (and their small scale variations) should be taken with care. Nevertheless, keeping in mind these limitations, static stress change modeling is extremely useful for the understanding of fault interaction processes as demonstrated by numerous previous studies.

In addition, the assumption of an elastic medium is an approximation to the real Earth that is usually invoked when investigating earthquake triggering and the interactions between faults and magmatic systems (e.g., refs^25,66–68^ among several others), and should be also appropriate for the addressed case studies. In particular, the assumed elastic parameters (see above) are compatible with those of igneous and metamorphic rocks that are expected to occur in the region (generally referred to as ‘basement’) located between the subduction thrust and the sedimentary wedge (Figs 2b, 3b,c; ref.^18^). The shallower part of the accretionary wedge is instead often characterized by thrust-top or forearc basins filled with weakly to moderately lithified sediments, which bear a stiffness obviously lower than that of the deeper rocks. Admittedly, an elastic dislocation modelling may be less accurate for such sediments of the upper sedimentary column, even though in some regions their thickness is rather limited (see geological sections in Fig. 3b,c). Nevertheless, the fact that these sediments are affected by thoroughgoing faults suggests that sediment compaction was large enough to achieve some shear strength, whereby the response to stress of similar sediments crossed by fault-controlled fluid pipes has been successfully modelled using an elastic and isotropic rheology^59^. On the other hand, the main target of this study is to probe the effect and magnitude of coseismic static stress changes generated by real megathrust earthquakes on fault-controlled fluid pathways, especially at depth, where the rocks the source fluids emerge from are expected to be elastic.

Static coseismic volumetric strain is defined as the ratio between the variation in volume of a body and its original volume, such that positive and negative values indicate dilation and contraction, respectively. Volumetric strain induces pore pressure change (Δ*p*), which, for a linear poroelastic material under undrained conditions, is given by the relation Δ*p* = −*BK*_u_ε_kk_/3, where *B* is the Skempton coefficient, *K*_u_ is the undrained bulk modulus, and ε_kk_ is the volumetric strain^51,60^. Notably, volumetric strain amplitude as small as 10^−6^ may be large enough to change permeability^48^.

## Supplementary information

Supporting Information
