# Supplementary material for: Seismic loading of fault-controlled fluid seepage systems by great subduction earthquakes
Source: Sci Rep. 2019 Aug 5;9:11332. doi: 10.1038/s41598-019-47686-4 (PMC6683289; doi:10.1038/s41598-019-47686-4)
Supplement: Supplementary file 1 — Supporting Information [file 41598_2019_47686_MOESM1_ESM.pdf]

Supporting Information

Seismic loading of fault-controlled fluid seepage systems by great subduction earthquakes

Marco Bonini[1,*]

[1]*CNR, Consiglio Nazionale delle Ricerche, Istituto di Geoscienze e Georisorse, Sede Secondaria Firenze, via G. La Pira 4, 50121, Firenze, Italy*

**corresponding author e-mails*: marco.bonini27@gmail.com; mbonini@igg.cnr.it

I provide further information on the coseismic static stress changes and volumetric static strain produced by different source fault models available for the M_w~7.9-8.2 1944 Tonankai earthquake, the M_w7.7 1992 Nicaragua earthquake, the M_w~8.5-8.7 2005 Nias earthquake, and the M_w7.6 2012 Costa Rica earthquake. Normal ($\Delta\sigma_n$) and shear ($\Delta\tau$) stress changes compose the Coulomb Failure Function[1,2,3,4,5,6], ΔCFF, which is used to determine whether slip on a source fault promotes failure on surrounding receiver faults, and this happens when ΔCFF is positive (see section Methods in main text). Normal stress change patterns ($\Delta\sigma_n$) obtained for the Tonankai and Costa Rica earthquakes are shown in two figures (Figs. S1-S2) as supplement to the main article. Normal stress change ($\Delta\sigma_n$), shear stress change ($\Delta\tau$) and Coulomb stress changes (ΔCFF) have been calculated using different finite-fault rupture models (some of which available on the online SRCMOD database[7]). The values of static stress changes have been resolved on specific fault-controlled fluid pathways (thrust or normal faults), and are reported in three tables (Tables S1-S3) (see also main text).

Fig. S1

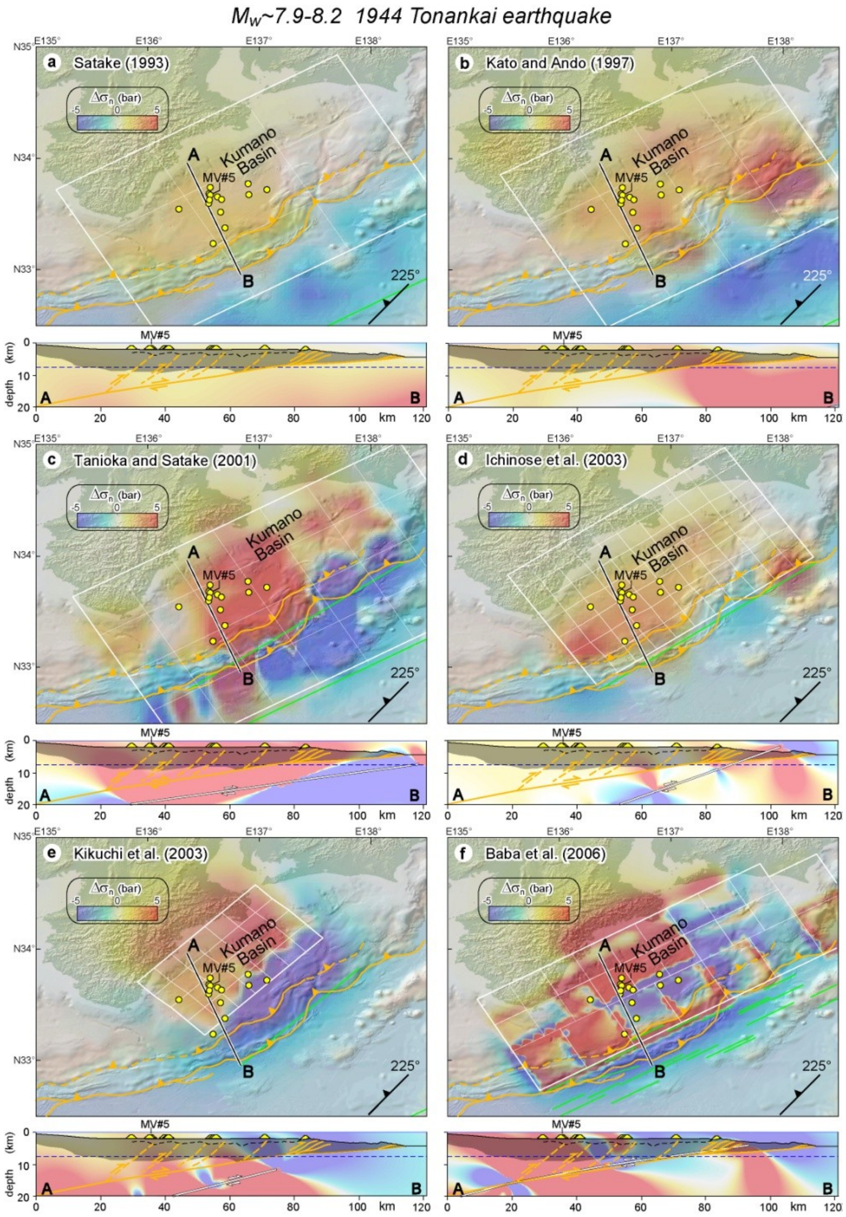

Figure S1. Normal stress changes ($\Delta\sigma_n$; bar, unclamping positive) produced by the M_w~7.9-8.2 1944 Tonankai earthquake considering different source fault models: (a) Satake[8], (b) Kato & Ando[9], (c) Tanioka & Satake[10], (d) Ichinose *et al.*[11], (e) Kikuchi *et al.*[12] and (f) Baba *et al.*[13]. Normal stress changes in vertical cross sections are superposed onto the Nankai sedimentary wedge[14], which is indicated by gray shading. A convenient range of stress change values (–5, 5 bar) has been arbitrarily chosen; note that in some models the calculated stresses may exceed this range. The thick white lines in sections indicate the seismogenic fault considered in the respective finite fault model. Note that the rupture fault is not captured in the cross section of panel (a), while in panel (b) it is intercepted only in the far lower right-hand side corner of the section. In horizontal sections stress is sampled at 7.5 km depth (dashed blue line in vertical cross sections). Symbols are as those in Fig. 2 of main text. This figure was generated using the Coulomb 3.4 software (https://earthquake.usgs.gov/research/software/coulomb) and Adobe Illustrator CS3 (https://www.adobe.com).

Fig. S2

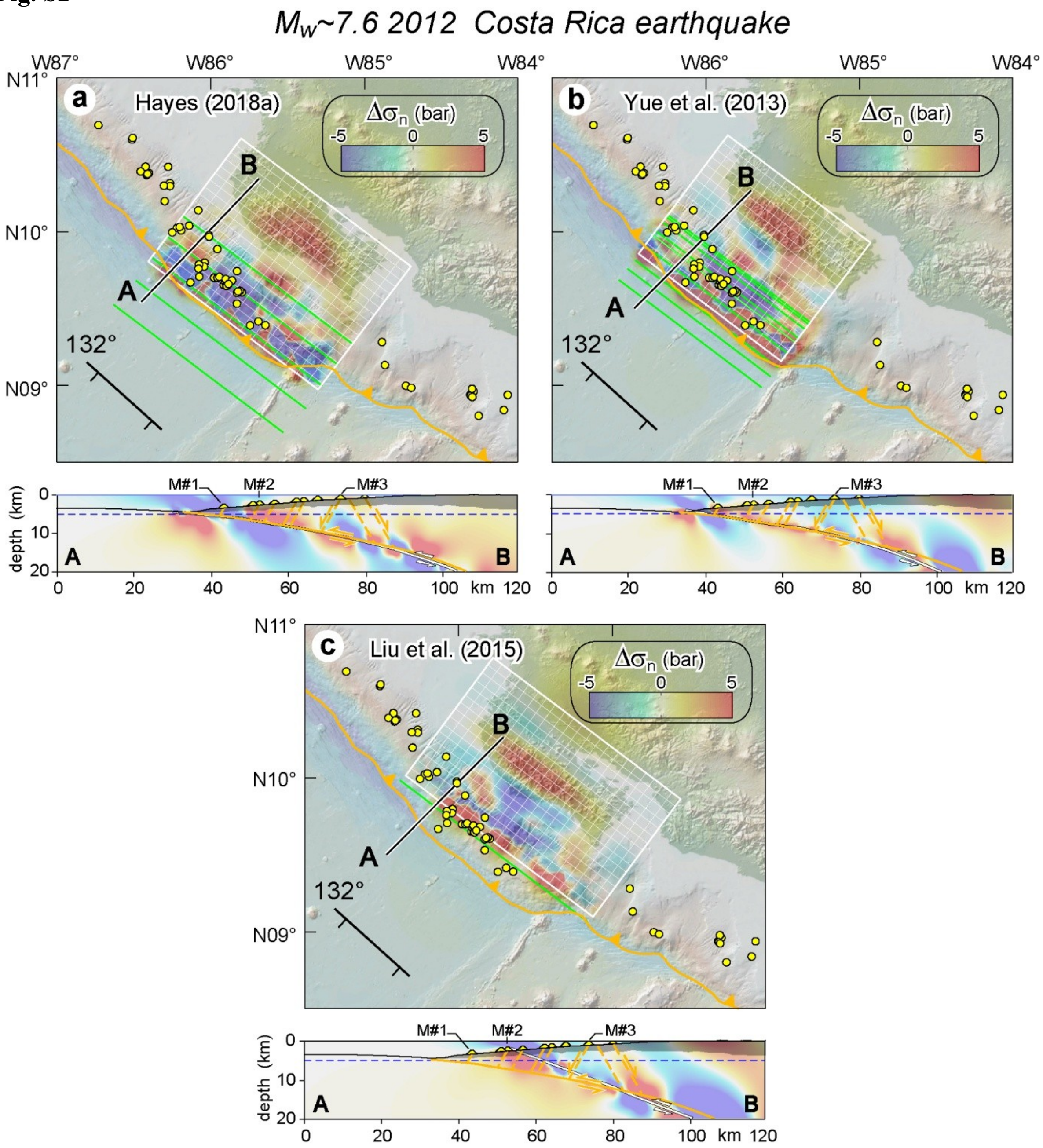

Figure S2. Normal stress changes ($\Delta\sigma_n$; bar, unclamping positive) produced by the M_w7.6 2012 Costa Rica earthquake considering different source fault models: (a) Hayes[16], (b) Yue *et al.*[17], and (c) Liu *et al.*[18] (symbols are as those in Fig. 2 of main text). Small yellow circles indicate different types of seafloor methane seeps. Normal stress changes in vertical cross sections are superposed onto the Costa Rica sedimentary wedge[19,20], which is indicated by gray shading. A convenient range of stress change values (–5, 5 bar) has been arbitrarily chosen; note that in some models the calculated stresses may exceed this range. The thick white lines in sections indicate the seismogenic source considered in the respective finite fault model. In horizontal sections stress is sampled at 5 km depth (dashed blue line in vertical cross sections). This figure was generated using the Coulomb 3.4 software (https://earthquake.usgs.gov/research/software/coulomb) and Adobe Illustrator CS3 (https://www.adobe.com).

Table S1. Static stress changes and static strain calculated from available source models of the 1944 Tonankai earthquake, as well as the M_w7.2 and M_w7.4 earthquakes of 05 September 2004. Normal stress change $\Delta\sigma_n$ (plain text), shear stress change $\Delta\tau$ (in parenthesis) and Coulomb Failure Function ΔCFF (bold) are sampled at various depths on a thrust fault (strike 225°, dip 40°, rake 90°) connecting MV#5 to the basal thrust décollement. For static strain, + indicates volumetric expansion, and − volumetric contraction. Source faults of 2004 earthquakes are based on focal mechanism solutions available from USGS (https://earthquake.usgs.gov/earthquakes/eventpage/usp000d3ka/executive; https://earthquake.usgs.gov/earthquakes/eventpage/usp000d3mb/executive), with fault dimensions following the relationships of Wells and Coppersmith[21].

M_w~7.9-8.2 1944 Tonankai earthquake						
Along thrust depth depth; Lat; Long	Satake[8]	Kato & Ando[9]	Tanioka & Satake[10]	Ichinose *et al.*[11]	Kikuchi *et al.*[12]	Baba *et al.*[13]
1.9 km; N33.676; E136.563	0.65 (-0.88) bar **-0.62 bar**	1.51 (-1.92) bar **-1.31 bar**	3.36 (-5.23) bar **-3.88 bar**	1.53 (-1.92) bar **-1.31 bar**	1.37 (-1.16) bar **-0.61 bar**	2.97 (-0.48) bar **0.71 bar**
5 km; N33.702; E136.538	0.76 (-0.97) bar **-0.67 bar**	1.40 (-1.70) bar **-1.14 bar**	3.31 (-3.31) bar **-1.98 bar**	1.27 (-1.77) bar **-1.26 bar**	2.26 (-1.37) bar **-0.47 bar**	10.98 (-4.02) bar **0.19 bar**
12 km; N33.755; E136.475	1.79 (-1.18) bar **-0.46 bar**	1.65 (-0.99) bar **-0.33 bar**	11.94 (-4.69) bar **0.08 bar**	0.82 (-1.12) bar **-0.79 bar**	3.17 (-4.74) bar **-3.48 bar**	26.26 (-18.1) bar **-7.58 bar**
15 km; N33.781; E136.452	2.37 (-1.31) bar **-0.36 bar**	1.69 (-0.73) bar **-0.06 bar**	5.84 (-9.37) bar **-7.03 bar**	0.92 (-0.78) bar **-0.41 bar**	4.29 (-6.61) bar **-4.90 bar**	38.52 (20.18) bar **35.59 bar**
Static strain	$+0.5\ 10^{-5}$	$+0.5\ 10^{-5}$	$+10^{-5}$	$+0.5\ 10^{-5}$	-10^{-5}	$+10^{-5}$
M_w7.2 and M_w7.4 2004 earthquakes						
Along thrust depth depth; Lat; Long	M_w7.2 - USGS 2004.09.05 10:07:07 UTC	M_w7.4 - USGS 2004.09.05 14:57:18 UTC	Cumulated sources	–	–	–
1.9 km; N33.676; E136.563	0.02 (0.02) bar **0.02 bar**	0.15 (-0.09) bar **-0.03 bar**	0.16 (-0.04) bar **0.02 bar**	–	–	–
5 km; N33.702; E136.538	0.08 (-0.04) bar **0.00 bar**	0.33 (-0.24) bar **-0.11 bar**	0.42 (-0.26) bar **-0.10 bar**	–	–	–
12 km; N33.755; E136.475	0.14 (-0.12) bar **-0.07 bar**	0.39 (-0.39) bar **-0.24 bar**	0.55 (-0.53) bar **-0.31 bar**	–	–	–
15 km; N33.781; E136.452	0.13 (-0.14) bar **-0.09 bar**	0.35 (-0.40) bar **-0.26 bar**	0.50 (-0.56) bar **-0.36ar**	–	–	–

Table S2. Static stress changes and static strain imparted by the M_w7.6 Costa Rica and M_w7.7 1992 Nicaragua earthquakes. Normal stress change $\Delta\sigma_n$ (plain text), shear stress change $\Delta\tau$ (in parenthesis) and Coulomb Failure Function ΔCFF (bold) are sampled on normal faults controlling specific mound structures located along section AB (Fig. 3b of main text) and CD (Fig. 3c of main text), respectively. Stress changes are calculated from available source models, and sampled at the sea bottom, 5 km depth, and near the basal thrust décollement considering a fault with strike 132°, dip 60°, and rake -90°. Shear stress change and Coulomb Failure Function are indicated in parenthesis and in bold, respectively. For static strain, + indicates volumetric expansion, and − volumetric contraction.

M_w 7.6 2012 Costa Rica earthquake			
Mound<br>Lat; Long; depth	Hayes[16]	Yue *et al.*[17]	Liu *et al.*[18]
M#1<br>N9.708; W86.074; 3.2 km<br><br>N9.703; W86.073; 5 km	 -4.50 (-0.97) bar<br>**-2.77 bar**<br>0.41 (2.00) bar<br>**2.16 bar**	 6.77 (19.20) bar<br>**21.91 bar**<br>33.58 (5.17) bar<br>**18.60 bar**	 0.55 (-1.01) bar<br>**-0.79 bar**<br>1.43 (0.01) bar<br>**0.58 bar**
M#2<br>N9.785; W86.074; 2.7 km<br><br>N9.774; W86.081; 5 km	 0.18 (0.43) bar<br>**-0.50 bar**<br>1.47 (-4.41) bar<br>**-3.83 bar**	 1.15 (1.13) bar<br>**1.59 bar**<br>4.46 (-1.21) bar<br>**0.58 bar**	 10.01 (2.66) bar<br>**6.66 bar**<br>4.15 (3.51) bar<br>**5.17 bar**
N9.765; W86.084; 7 km	6.61 (1.55) bar<br>**4.19 bar**	-0.56 (-6.81) bar<br>**-7.04 bar**	2.79 (2.82) bar<br>**3.93 bar**
M#3<br>N9.967; W86.012; 1.2 km<br><br>N9.953; W86.023; 5 km	 1.39 (1.09) bar<br>**1.64 bar**<br>-0.67 (-0.15) bar<br>**-0.42 bar**	 -1.94 (-1.36) bar<br>**-2.13 bar**<br>-1.51 (-1.84) bar<br>**-2.45 bar**	 -1.21 (-0.87) bar<br>**-1.35 bar**<br>-1.62 (-0.32) bar<br>**-1.35 bar**
N9.934; W86.039; 10 km	3.68 (-2.07) bar<br>**-0.60 bar**	2.10 (-1.93) bar<br>**-1.09 bar**	2.62 (-0.41) bar<br>**0.64 bar**
Static strain	$+10^{-5}$	$+10^{-6}$	$-0.5\ 10^{-5}$
M_w7.7 1992 Nicaragua earthquake			
Mound<br>Lat; Long; depth	Hayes[22]	−	−
M#4<br>N11.185; W87.200; 1.5 km	 15.87 (8.26) bar<br>**14.60 bar**	−	−
N11.172; W87.212; 5 km<br><br>N11.160; W87.223; 8 km	19.87 (7.73) bar<br>**15.68 bar**<br>32.03 (52.04) bar<br>**64.86 bar**	−	−
M#5<br>N11.203; W87.156; 1.2 km<br><br>N11.189; W87.168; 5 km	 11.16 (5.42) bar<br>**9.88 bar**<br>19.46 (6.52) bar<br>**14.30 bar**	−	−
N11.176; W87.175; 9 km	7.00 (9.50) bar<br>**12.30 bar**	−	−
M#6<br>N11.239; W87.133; 0.9 km<br><br>N11.223; W87.144; 5 km	 7.93 (4.87) bar<br>**8.04 bar**<br>11.44 (3.63) bar<br>**8.51 bar**	−	−
N11.203; W87.163; 10 km	42.84 (44.78) bar<br>**61.92 bar**	−	−
Static strain	$+10^{-5}$	−	−

Table S3. Static stress changes and static strain imparted by the M_w8.5-8.7 2005 Nias earthquake. Normal stress change $\Delta\sigma_n$ (plain text), shear stress change $\Delta\tau$ (in parenthesis) and Coulomb Failure Function ΔCFF (bold) are sampled on thrust faults controlling specific seepage structures. Seepage structures consist of mud volcanoes (MV) and mud diapirs (MD) exposed on Nias and Simeulue islands[23,24], as well as an offshore methane cold seep systems exhibiting extensive buildups of authigenic carbonate deposits[25] (MS). Stress changes are calculated from available source models, and sampled at surface (or at sea floor) and at depth near the basal subduction thrust considering a fault with strike 325°, dip 40°, and rake 90°. The basal subduction thrust has been speculatively assumed as that of Singh *et al.*[26]. The latter shows, however, some difference with the geometry of source faults used in the different finite fault models (see Fig. 4 of main text). For this reason, stress changes have also been calculated near the intersection with the source fault of each finite fault model. Static strain (+ indicates volumetric expansion, and − volumetric contraction) is estimated beneath the seepage structures situated along section AB in Figure 4 of main text.

M_w8.5-8.7 2005 Nias earthquake				
Mud volcano/diapir/seep<br>Lat; Long; depth	Shao & Ji[27]	Konca *et al.*[28]	Yatimantoro & Tanioka[29]	Hayes[30]
MV#1 Nias Island				
N1.435; E97.456; 0 km	6.59 (-7.85) bar<br>**-5.22 bar**	1.56 (-1.86) bar<br>**-1.24 bar**	4.65 (-5.54) bar<br>**-3.68 bar**	1.52 (-1.81) bar<br>**-1.20 bar**
N1.478; E97.488; 5 km	10.73 (-12.38) bar<br>**-8.08 bar**	6.21 (-5.86) bar<br>**-3.38 bar**	7.98 (-8.66) bar<br>**-5.47 bar**	0.65 (-1.21) bar<br>**-0.95 bar**
N1.524; E97.518; 10 km	9.03 (-15.80) bar<br>**-12.19 bar**	13.43 (-10.50) bar<br>**-5.13 bar**	17.12(-13.80) bar<br>**-6.95 bar**	1.99 (-2.48) bar<br>**-1.69 bar**
N1.565; E97.548; 15 km	4.40 (-10.85) bar<br>**-9.09 bar**	19.29 (-15.57) bar<br>**-7.85 bar**	36.71 (-13.13) bar<br>**1.55 bar**	1.38 (-4.41) bar<br>**-3.86 bar**
N1.610; E97.579; 20 km	7.89 (-8.97) bar<br>**-5.81 bar**	22.04 (-17.21) bar<br>**-8.39 bar**	79.10 (-25.21) bar<br>**6.42 bar**	-0.92 (-3.56) bar<br>**-3.93 bar**
Near model source fault	-29.20 (12.05) bar<br>**0.36 bar**	13.54 (-15.84) bar<br>**-10.43 bar**	-4.72 (0.44) bar<br>**-1.45 bar**	-16.02 (5.06) bar<br>**-1.35 bar**
MV#2 Nias Island				
N1.357; E97.542; 0 km	8.17 (-9.74) bar<br>**-6.47 bar**	0.41 (-0.49) bar<br>**-0.33 bar**	6.32 (-7.53) bar<br>**-5.00 bar**	0.35 (-0.42) bar<br>**-0.28 bar**
N1.401; E97.574; 5 km	11.51 (-14.46) bar<br>**-9.85 bar**	3.52 (-4.05) bar<br>**-2.65 bar**	7.38 (-8.43) bar<br>**-5.48 bar**	-0.13 (-0.36) bar<br>**-0.41 bar**
N1.445; E97.605; 10 km	11.97 (-16.30) bar<br>**-11.51 bar**	6.97 (-8.04) bar<br>**-5.25 bar**	20.86 (-10.40) bar<br>**-2.05 bar**	4.09 (-2.00)bar<br>**-0.36 bar**
N1.488; E97.635; 15 km	16.18 (-12.09) bar<br>**-5.61 bar**	9.58 (-11.36) bar<br>**-7.53 bar**	49.76 (-11.84) bar<br>**8.06 bar**	8.25 (-4.13) bar<br>**-0.83 bar**
N1.531; E97.665; 20 km	6.22 (-17.64) bar<br>**-15.15 bar**	13.37 (-12.91) bar<br>**-7.56 bar**	52.14 (-38.54) bar<br>**-17.68 bar**	11.30 (-4.92) bar<br>**-0.40 bar**
Near model source fault	-40.97 (25.04) bar<br>**8.65 bar**	6.76 (-12.74) bar<br>**-10.03 bar**	6.11 (-5.73) bar<br>**-3.29 bar**	-18.10 (0.88) bar<br>**-6.37 bar**
MD#1 Nias Island				
N1.236; E97.389; 0 km	10.91 (-13.00) bar<br>**-8.64 bar**	4.83 (-5.75) bar<br>**-3.82 bar**	5.30 (-6.31) bar<br>**-4.19 bar**	8.94 (-10.66) bar<br>**-7.08 bar**
N1.262; E97.436; 5 km	13.22 (-16.17) bar<br>**-10.88 bar**	0.89 (-2.80) bar<br>**-2.44 bar**	8.57 (-3.872) bar<br>**-0.44 bar**	0.59 (-4.42) bar<br>**-4.18 bar**
N1.299; E97.472; 10 km	14.95 (-20.22) bar<br>**-14.24 bar**	3.07 (-3.53) bar<br>**-2.31 bar**	20.46 (-8.16) bar<br>**0.02 bar**	-2.90 (-1.18) bar<br>**-2.34 bar**
N1.338; E97.511; 15 km	34.01 (-20.26) bar<br>**-6.65 bar**	6.92 (-8.51) bar<br>**-5.74 bar**	35.48 (-18.65) bar<br>**-4.46 bar**	2.22 (-0.77) bar<br>**0.12 bar**
N1.376; E97.549; 20 km	30.72 (-47.30) bar<br>**-35.01 bar**	8.58 (-12.37) bar<br>**-8.94 bar**	52.55 (-34.24) bar<br>**-13.22 bar**	11.75 (-5.14) bar<br>**-0.44 bar**
Near model source fault	-120.17 (67.78) bar<br>**19.71 bar**	11.05 (-28.24) bar<br>**-23.82 bar**	111.04 (-41.03) bar<br>**3.38 bar**	24.46 (-16.63) bar<br>**-6.85 bar**
MD#2 Nias Island				
N1.052; E97.572; 0 km	9.26 (-11.03) bar<br>**-7.33 bar**	-1.73 (2.07) bar<br>**1.37 bar**	3.77 (-4.49) bar<br>**-2.98 bar**	9.25 (-11.03) bar<br>**-7.33 bar**

N1.090; E97.609; 5 km	13.60 (-13.97) bar<br>**-8.53 bar**	-2.55 (2.25) bar<br>**1.23 bar**	1.84 (-3.11) bar<br>**-2.38 bar**	-0.86 (-3.61) bar<br>**-3.96 bar**
N1.129; E97.645; 10 km	25.93 (-19.99) bar<br>**-9.62 bar**	2.91 (-0.63) bar<br>**0.54 bar**	-1.62 (-2.13) bar<br>**-2.78 bar**	-2.79 (-0.61) bar<br>**-1.72 bar**
N1.167; E 97.685; 15 km	38.52 (-38.57) bar<br>**-23.17 bar**	8.24 (-4.24) bar<br>**-0.95 bar**	-4.86 (-2.83) bar<br>**-4.77 bar**	4.52 (-1.95) bar<br>**-0.15 bar**
N1.207; E97.720; 20 km	30.91 (-52.94) bar<br>**-40.58 bar**	14.52 (-7.35) bar<br>**-1.54 bar**	-7.09 (-5.65) bar<br>**-8.49 bar**	17.03 (-8.52) bar<br>**-1.71 bar**
Near model source fault	-160.41 (-7.74) bar<br>**-71.90 bar**	0.58 (-10.08) bar<br>**-9.85 bar**	39.24 (-51.88) bar<br>**-36.19 bar**	107.96 (-82.14) bar<br>**-38.96 bar**
MS#1 offshore				
N2.563; E96.757; 1.4 km	-1.23 (1.39) bar<br>**0.90 bar**	3.12 (-2.56) bar<br>**-1.31 bar**	0.27 (0.78) bar<br>**0.89 bar**	-1.72 (1.28) bar<br>**0.59 bar**
N2.589; E96.788; 5 km	-0.10 (2.14) bar<br>**2.10 bar**	6.38 (-5.06) bar<br>**-2.51 bar**	4.27 (-0.73) bar<br>**0.98 bar**	0.02 (0.25) bar<br>**0.26bar**
N2.618; E96.833; 10 km	2.65 (2.05) bar<br>**3.11 bar**	6.08 (-6.46) bar<br>**-4.03 bar**	8.16 (-1.58) bar<br>**1.68 bar**	6.56 (-2.83) bar<br>**-0.20 bar**
N2.646; E96.879; 15 km	2.07 (1.22) bar<br>**2.05 bar**	2.37 (-5.94) bar<br>**-4.99 bar**	9.22 (-2.20) bar<br>**1.49 bar**	12.91 (-6.99) bar<br>**-1.83 bar**
N2.679; E96.922; 20 km	11.72 (3.18) bar<br>**7.87 bar**	-1.49 (-3.70) bar<br>**-4.30 bar**	4.90 (-2.92) bar<br>**-0.96 bar**	15.09 (-11.13) bar<br>**-5.10 bar**
Near model source fault	-24.91 (34.15) bar<br>**24.19 bar**	-4.33 (1.26) bar<br>**-0.47 bar**	13.11 (-5.93) bar<br>**-0.69 bar**	-0.37 (-0.91) bar<br>**-1.06 bar**
MV#3 Simeulue Island				
N2.370; E96.403; 0 km	-0.89 (1.06) bar<br>**0.71 bar**	11.45 (-13.65) bar<br>**-9.01 bar**	-7.88 (9.39) bar<br>**6.24 bar**	-2.50 (2.98) bar<br>**1.98 bar**
N2.413; E96.434; 5 km	0.15 (-1.15) bar<br>**-1.09 bar**	0.25 (-6.34) bar<br>**-6.24 bar**	0.12 (5.98) bar<br>**6.03 bar**	9.07 (-2.31) bar<br>**1.32 bar**
N2.458; E96.467; 10 km	-4.14 (-2.01) bar<br>**-3.67 bar**	6.26 (-5.94) bar<br>**-3.44 bar**	2.15 (4.40)bar<br>**5.26 bar**	6.23 (-3.32) bar<br>**-0.83 bar**
N2.500; E96.497; 15 km	-6.56 (2.49) bar<br>**-0.13 bar**	23.04 (-21.13) bar<br>**-11.91 bar**	-10.84 (0.83) bar<br>**-3.50 bar**	-4.82 (-1.70) bar<br>**-3.63 bar**
N2.546; E96.529; 20 km	-12.62 (3.63) bar<br>**-1.42 bar**	16.97 (-33.75) bar<br>**-26.96 bar**	18.61 (2.21) bar<br>**9.65 bar**	-12.46 (5.66) bar<br>**0.67 bar**
Near model source fault	-7.56 (-0.45) bar<br>**-3.47 bar**	-15.79 (-37.71) bar<br>**-44.03 bar**	13.07 (-7.10) bar<br>**-1.88 bar**	34.54 (-3.98) bar<br>**9.84 bar**
MV#4 Simeulue Island				
N2.397; E96.371; 0 km	-0.96 (1.14) bar<br>**0.76 bar**	13.27 (-15.68) bar<br>**-10.51 bar**	-8.37 (9.98) bar<br>**6.63 bar**	-4.15 (4.95) bar<br>**3.29 bar**
N2.447; E96.389; 5 km	3.01 (-1.78) bar<br>**-0.58 bar**	-0.91 (-7.59) bar<br>**-7.96 bar**	-0.97 (6.57) bar<br>**6.19 bar**	8.92 (-0.68) bar<br>**2.89 bar**
N2.495; E96.406; 10 km	0.95 (-4.63) bar<br>**-4.25 bar**	1.94 (-4.84) bar<br>**-4.07 bar**	2.29 (6.34) bar<br>**7.26 bar**	9.61 (-1.57) bar<br>**2.27 bar**
N2.548; E96.425; 15 km	-5.51 (-1.86) bar<br>**-4.06 bar**	38.76 (-16.11) bar<br>**-0.61 bar**	0.14 (7.61) bar<br>**7.67 bar**	-0.04 (2.34) bar<br>**2.32 bar**
N 2.588; E96.438; 20 km	-6.08 (-0.02) bar<br>**-2.46 bar**	57.36 (-56.51) bar<br>**-33.56 bar**	-92.54 (0.15) bar<br>**-36.87 bar**	-12.92 (9.93) bar<br>**4.76 bar**
Near model source fault	-7.35 (-0.21) bar<br>**-3.15 bar**	-9.54 (-53.49) bar<br>**-57.31 bar**	11.59 (-7.67) bar<br>**-3.03 bar**	35.03 (-17.50) bar<br>**-3.49 bar**
Static strain	$+10^{-5}$	$+0.5\ 10^{-5}$	$+0.5\ 10^{-5}$	$+0.5\ 10^{-5}$

References

1. Reasenberg, P.A. & Simpson, R.W. Response of regional seismicity to the static stress change produced by the Loma Prieta earthquake. *Science* **255**, 1687–1690 (1992).
2. Stein, R.S., King, G.C.P. & Lin, J. Change in failure stress on the Southern San Andreas fault system caused by the 1992 magnitude = 7.4 Landers earthquake. *Science* **258**, 1328–1332 (1992).
3. King, G.C.P., Stein, R.S. & Lin, J. Static stress changes and the triggering of earthquakes. *Bull. Seismol. Soc. Am.* **84**, 935–953 (1994).
4. Harris, R.A. Introduction to special section: stress triggers, stress shadows and implications for seismic hazard. *J. Geophys. Res.* **103**, 24,347–24,358 (1998).
5. Stein, R.S. The role of stress transfer in earthquake occurrence. *Nature* **402**, 605–609 (1999).
6. Kilb, D., Gomberg, J. & Bodin, P. Aftershock triggering by complete Coulomb stress changes. *J. Geophys. Res.* **107**, 2060 (http://dx.doi.org/10.1029/2001JB000202; 2002).
7. Mai, M. & Thingbaijam, K.K.S. SRCMOD: An online database of finite-fault rupture models. *Seismological Research Letters* **85**, 1348-1357 (2014).
8. Satake, K. Depth distribution of coseismic slip along the Nankai Trough, Japan, from joint inversion of geodetic and tsunami data. *J. Geophys. Res.* **98**, 4553-4565 (1993).
9. Kato, T. & Ando, M. Source mechanisms of the 1944 Tonankai and 1946 Nankaido earthquakes: Spatial heterogeneity of rise times. *Geophys. Res. Lett.* **24**, 2055-2058 (1997).
10. Tanioka, Y. & Satake. K. Detailed coseismic slip distribution of the 1944 Tonankai earthquake estimated from tsunami waveforms. *Geophys. Res. Lett.* **28**,1075-1078 (2001).
11. Ichinose, G.A., Thio, H.K., Somerville, P.G., Sato, T. & Ishii, T. Rupture process of the 1944 Tonankai earthquake (Ms 8.1) from the inversion of teleseismic and regional seismograms. *J. Geophys. Res.* **108**, B2497 (2003).
12. Kikuchi, M., Nakamura, M. & Yoshikawa, K. Source rupture processes of the 1944 Tonankai earthquake and the 1945 Mikawa earthquake derived from low-gain seismograms. *Earth Planets Space* **55**, 159–172 (2003).
13. Baba, T., Cummins, P.R., Hori, T. & Kaneda, Y. High precision slip distribution of the 1944 Tonankai earthquake inferred from tsunami waveforms: Possible slip on a splay fault. *Tectonophysics* **426**, 119–134 (2006).
14. Park, J.O., Kaneda, Y., Tsuru, T., Kodaira, S. & Cummins, P.R. Splay fault branching along the Nankai subduction zone. *Science* **297**, 1157-1160 (2002).
15. Marcaillou, B. *et al.* Seismogenic zone temperatures and heat-flow anomalies in the To-nankai margin segment based on temperature data from IODP expedition 333 and thermal model. *Earth and Planetary Science Letters* **349-350**, 171–185 (2012).
16. Hayes, G. Finite fault model of the September 05, 2012 Mw 7.6 Costa Rica earthquake. Contributed by USGS National Earthquake Information Center, last updated 2018.10.17; https://earthquake.usgs.gov/earthquakes/eventpage/usp000jrsw/executive (2018a).
17. Yue, H. *et al.* The 5 September 2012 Nicoya, Costa Rica Mw 7.6 earthquake rupture process from joint inversion of high-rate GPS, strong-motion, and teleseismic P wave data and its relationship to adjacent plate boundary interface properties. *J. Geophys. Res.* **118,** 5453–5466 (2013).

18. Liu, C. *et al.* Rupture processes of the 2012 September 5 Mw 7.6 Nicoya, Costa Rica earthquake constrained by improved geodetic and seismological observations. *Geophys. J. Int.* **203**, 175–183 (2015).

19. Ranero, C.R. *et al.* Hydrogeological system of erosional convergent margins and its influence on tectonics and interplate seismogenesis. *Geochem. Geophys. Geosyst.* **9**, Q03S04 (2008).

20. Ranero, C.R. *et al.* A cross section of the convergent Pacific margin of Nicaragua. *Tectonics* **19**, 335-357 (2000).

21. Wells, D.L. & Coppersmith, K.J. New empirical relationships among magnitude, rupture length, rupture width, rupture area, and surface displacement. *Bull. Seismol. Soc. Am.* **84**, 974–1002 (1994).

22. Hayes, G. Finite fault model of the September 02, 1992 Mw 7.7 Nicaragua earthquake. Contributed by USGS National Earthquake Information Center, last updated 2018.10.17; https://earthquake.usgs.gov/earthquakes/eventpage/usp0005ddn/executive (2018b).

23. Barber, A.J. The origin of mélanges: Cautionary tales from Indonesia. *Journal of Asian Earth Sciences* **76**, 428–438 (2013).

24. Aribowo, S. *et al.* Complex deformation in Simeulue Island, Sumatra: interplay between structure and diapirism. *Ris. Geo. Tam* **24**, 131-144 (2014).

25. Siegert, M., Krüger, M., Teichert, B., Wiedicke, M. & Schippers, A. Anaerobic oxidation of methane at a marine methane seep in a forearc sediment basin off Sumatra, Indian Ocean. *Frontiers in Microbiology* **2**, Article 249 (2011).

26. Singh, S.C. *et al.* Seismic evidence for broken oceanic crust in the 2004 Sumatra earthquake epicentral region. *Nature Geoscience* **1**, 777-781 (2008).

27. Shao, G. & Ji, C. Preliminary Result of the Mar 28, 2005 Mw 8.68 Nias earthquake. National Earthquake Information Center (NEIC) of United States Geological Survey. http://www.geol.ucsb.edu/faculty/ji/big_earthquakes/2005/03/smooth/nias.html (2005).

28. Konca, O. *et al.* Rupture kinematics of the 2005 Mw 8.6 Nias–Simeulue earthquake from the joint inversion of seismic and geodetic data. *Bulletin of the Seismological Society of America* **97**, S307–S322 (2007).

29. Yatimantoro, T. & Tanioka, Y. Determination of slip distribution of the 28 March 2005 Nias earthquake using joint inversion of tsunami waveform and GPS data. *Bulletin of IISEE* **47**, 115-120 (2013).

30. Hayes, G. Finite fault model of the March 28, 2005 Mw 8.6 northern Sumatra earthquake. Contributed by USGS National Earthquake Information Center, last updated 2018.10.17; https://earthquake.usgs.gov/earthquakes/eventpage/official20050328160936530_30/executive (2018c).
